# Sustainable therapies by engineered bacteria

**DOI:** 10.1111/1751-7915.12778

**Published:** 2017-07-11

**Authors:** Beatriz Álvarez, Luis Ángel Fernández

**Affiliations:** ^1^ Department of Microbial Biotechnology Centro Nacional de Biotecnología, Consejo Superior de Investigaciones Científicas (CNB‐CSIC) Campus UAM Cantoblanco 28049 Madrid Spain

## Abstract

The controlled *in situ* delivery of biologics (e.g. enzymes, cytokines, antibodies) by engineered bacteria of our microbiome will allow the sustainable production of these complex and expensive drugs locally in the human body, overcoming many of the technical and economical barriers currently associated with the global use of these potent medicines. We provide examples showing how engineered bacteria can be effective treatments against multiple pathologies, including autoimmune and inflammatory diseases, metabolic disorders, diabetes, obesity, infectious diseases and cancer, hence contributing to achieve the Global Sustainable Goal 3: ensure healthy lives and promote well‐being for all at all ages.

## Biologics and microbial engineering

The pharmaceutical industry is moving from small chemical drugs towards more complex and potent biological drugs, or ‘biologics’, therapeutics based on large macromolecules, such as enzymes, growth factors, cytokines and antibodies. The large size of biologics allows a higher level of specificity, which reduces the non‐target side‐effects. In addition, they can be engineered by rational design and molecular evolution to improve their functional properties (i.e. specificity, half‐life, activity). As a consequence, biologics are already dominating the list of top drugs providing the highest incomes to the pharmaceutical industry (PharmaCompass, 2016). At present, antibodies (Abs) are the biologics showing the highest increase, with over 50 molecules already approved for treatment of major diseases such as cancer, autoimmune diseases and inflammation, and whose turnover exceeds 75 billion euros per year (Ecker *et al*., 2015; Reichert, 2016).

However, the downside of biologics is their elevated cost, which is related to high investments in their development phase, and the technical difficulties of their large‐scale production, purification and distribution of these complex molecules, as they are in general more labile and short lived than small chemical drugs. The elevated cost of biologics is having a negative impact on the budget of the national health systems in developed countries, and represents a strong barrier for their use in low‐income countries. Hence, the future growth and global access of these potent medicines of the XXI century will be compromised unless novel technologies help to solve these bottlenecks. In this context, microbial biotechnology already provides important solutions at various levels, such as increasing production yields in bioreactors using microorganisms (i.e. yeasts, bacteria), reducing the time and cost associated with the slow growth of mammalian cells, and assisting on the optimization and engineering of biologics thanks to the high cloning capacity of microorganisms and the efficient screening methods of microbial libraries (Hoogenboom, 2005; Galan *et al*., 2016). In addition to these conventional technologies, microbial engineering is developing innovative approaches to produce and deliver complex biologics *in situ*, within the body, ideally in a controlled and programmable way. These are based on the engineering of probiotic and commensal bacteria from human microbiota, using both classical genetic engineering and synthetic biology tools, to generate therapeutic bacteria that produce the desired biologic (Cameron *et al*., 2014; Piñero‐Lambea *et al*., 2015a). Here, we briefly discuss the potential of the use of engineered bacteria for the treatment of human diseases in an affordable and effective way that will help to accomplish the objectives of Sustainable Development Goal 3: ensuring healthy lives and promoting the well‐being for all at all ages. The use of engineered bacteria against cancer will not be discussed here because it is the topic of a different article in this issue.

## Engineering bacteria and the microbiome

The microbial population (microbiota or microbiome) that colonizes the skin and human mucosal surfaces, especially the gastrointestinal tract (GIT), is essential to keep the health status of the body. Many studies have shown the relationship between microbiome dysbiosis and disease, such as infections, inflammation, allergy, asthma, obesity, cancer and even neurological disorders (Sekirov *et al*., 2010; Carding *et al*., 2015; Li *et al*., 2016; Foo *et al*., 2017). Therefore, it is reasonable to think that interventions on the microbiome with specific microorganisms will allow the development of therapies for such diseases (Lemon *et al*., 2012). Effective therapies require a defined, reproducible therapeutic composition and known mechanism of action. This can be achieved by rationale engineering of commensal bacteria found in the microbiome to confer upon them specific properties to efficiently combat the disease, such as resistance to stress conditions, colonization of a desired niche, controlled delivery of therapeutic biologics and biocontainment. The selection of the bacterial strain chassis to develop the designed engineering is crucial for an effective therapy. The chassis strain should be non‐pathogenic, amenable to genetic manipulation and, ideally, naturally adapted to live in the site where the therapeutic action is needed. Importantly, there is ample experience in the last century on the administration into patients of natural bacterial strains of the microbiota from healthy individuals, called ‘probiotics’. These intentional administrations with natural bacteria have been mostly performed with strains of lactic acid bacteria (LAB) (e.g. *Lactobacillus, Bifidobacterium*) but also with *Escherichia coli* strains, such as *E. coli* Nissle 1917 (EcN; Behnsen *et al*., 2013; Ou *et al*., 2016; Sonnenborn, 2016). These strains usually have limited therapeutic effects by themselves, but they have clearly proven to be safe, and thus represent excellent chassis for synthetic biology engineering (Chua *et al*., 2017).

## Engineered bacteria against immunological and metabolic diseases

Given the extensive use of LAB as probiotics in foods, it is not surprising that early work on engineering therapeutic bacteria involved LAB and diseases of the GIT, like inflammatory bowel diseases (IBDs; Xavier and Podolsky, 2007; Rescigno, 2014; Chu *et al*., 2016). A *Lactococcus lactis* strain was engineered to locally deliver the anti‐inflammatory cytokine IL‐10 in the GIT (Steidler *et al*., 2000). This strain went successfully through a phase 1 clinical study in patients (Braat *et al*., 2006). The strain was shown to be safe and biologically contained, but was not effective in treating the disease in the subsequent phase 2a clinical study. Nevertheless, it is noteworthy that it was the first engineered microbial strain tested in clinical trials and the results have been encouraging for other genetically modified *L. lactis* strains that went into clinical trials (Bermudez‐Humaran *et al*., 2013). The company Interxon (https://www.dna.com/) is developing biotherapeutics based on modified *L. lactis* to treat inflammatory and autoimmune diseases, as well as other illnesses associated with cancer chemotherapy. LAB have been also engineered to induce mucosal immune tolerance of causative antigens in food allergies, such coeliac disease (Huibregtse *et al*., 2009). Induction of tolerance has also been applied to downregulate autoimmune diseases like type 1 diabetes (T1D), in which self‐antigens from pancreatic β‐cells are recognized by the immune system (Takiishi *et al*., 2012; Robert *et al*., 2014). In addition, engineered bacteria can modulate levels of cytokines outside the GIT, preventing cardiovascular pathologies (Jing *et al*., 2011) and encephalomyelitis (Rezende *et al*., 2013). Administration of engineered bacteria in the gut can also protect from metabolic disorders. For instance, LAB producing the hormone glucagon‐like peptide (GLP)‐1 was able to induce insulin production in gut epithelial cells in a glucose‐responsive manner, thereby reducing hyperglycaemia (Duan *et al*., 2015).

Engineering of a different probiotic chassis, EcN, has also produced relevant strains with therapeutic effects against IBDs (Whelan *et al*., 2014) and against obesity (Chen *et al*., 2014). The company Synlogic (http://www.synlogictx.com/) is engineering EcN as a microbial chassis for development of human therapeutics (Synthetic Biotic™) against IBDs, cancer and metabolic deficiencies, such as urea cycle disorder and phenylketonuria (http://www.synlogictx.com/pipeline/pipeline/).

## Engineered bacteria against infectious diseases

The above‐mentioned strains target non‐communicable diseases, including rare metabolic diseases, and may help to provide access to affordable biologic medicines for all, which is an important objective of Sustainable Development Goal 3. But also, a major health goal is the treatment of communicable infectious diseases, especially those acting on children (e.g. diarrhoea), or being global epidemics (e.g. HIV, tuberculosis). Engineered bacteria can also help in these objectives. Different strategies have been investigated against infectious diseases, including toxin neutralization, blocking pathogen adhesion or interfering with quorum sensing (QS) signals (Goh *et al*., 2012; Hwang *et al*., 2016). There are examples of engineered strains against major diarrhoeal diseases, including bacteria such as *Vibrio cholera* (Duan and March, 2010) and viruses like rotaviruses, a major cause of severe diarrhoea in children under 5 years (Pant *et al*., 2006; Álvarez *et al*., 2015). LAB commensals in human vagina were also engineered to produce an antiviral activity against HIV, which showed protection against simian HIV infection in macaques (Lagenaur *et al*., 2011). In another elegant example, EcN was engineered to produce bacteriocins and swim towards the pathogen in response to QS signals of *Pseudomonas aeruginosa* (Hwang *et al*., 2014), and the resulting construct shown to exhibit antimicrobial activity against this pathogen in the gut of mouse models (Hwang *et al*., 2017). The above examples also illustrate that therapeutic bacteria can be engineered to be extremely selective against a specific pathogen, reducing the use (or abuse) of antibiotics, hence reducing the spread of antibiotic resistances in nature (e.g. tuberculosis). Importantly, these approaches are not only of use in humans to prevent infection, but are also of great interest for interventions in the microbiota of animal reservoirs or insect vectors of human disease (Hurwitz *et al*., 2012; Foo *et al*., 2017). Thus, major communicable diseases (e.g. malaria) could be prevented at an earlier stage, in the animal reservoir or insect, prior to the actual human infection.

## Synthetic biology engineering

Synthetic biology is providing modular parts and gene circuits that can be used to program the designed bacterial chassis to precisely control the expression and delivery of therapeutic proteins (Huh *et al*., 2013; Reeves *et al*., 2015; Ruano‐Gallego *et al*., 2015), the adhesion of the engineered bacteria to target cells (Piñero‐Lambea *et al*., 2015b) or modify their chemotactic behaviour (Hwang *et al*., 2014). Bacteria have also been engineered with gene circuits that respond to disease signals in the gut (Riglar *et al*., 2017). In addition, synthetic biology is developing tools to manipulate bacteria that are very abundant in the human microbiome, like the genus *Bacteroides* (Lim *et al*., 2017).

## Concluding remarks

In the near future, it will be possible to program bacteria like a nanomachine to treat a pathology. The engineered bacteria will act specifically and locally, which reduces the probability of side‐effects. These microorganisms will be controlled so that they will respond to stimuli indicating disease and can be eliminated once the pathology is resolved. The engineered bacterial strains will be able to combat specific pathogens, contributing to overcoming the growing problem of antibiotic resistance. The designed therapies will be also economically viable. The production will be inexpensive because it would only require the growth of the microorganism without complex purification steps. The distribution can also be facilitated because many bacteria can be freeze‐dried without affecting their viability, so no cold chain will be needed. These properties will definitely help to achieve Sustainable Development Goal 3, reducing major diarrhoeal diseases and epidemics like HIV and malaria, fighting against antibiotic resistance and combatting important autoimmune and metabolic diseases with affordable, safe and potent medicines for all.

## Conflict of interest

The authors declare that they have no conflict of interest.
